# Exploring health professionals' views on the depiction of conversational agents as health professionals: a qualitative descriptive study

**DOI:** 10.3389/fdgth.2025.1590514

**Published:** 2025-09-22

**Authors:** A. Luke MacNeill, Lillian MacNeill, Alison Luke, Shelley Doucet

**Affiliations:** ^1^ Centre for Research in Integrated Care, University of New Brunswick, Saint John, NB, Canada; ^2^ Department of Nursing and Health Sciences, University of New Brunswick, Saint John, NB, Canada

**Keywords:** conversational agents, chatbots, health care, health professionals, qualitative, interview, views

## Abstract

**Background:**

Some health care conversational agents (HCCAs) are designed to simulate health professionals in terms of their presentation or appearance. Research suggests that the public has favorable views toward the depiction of HCCAs as health professionals, but the views of health professionals are less clear. We conducted a qualitative descriptive study to learn more about health professionals' views on this topic.

**Methods:**

Physicians, nurses, and regulated mental health professionals were recruited using web-based methods. Participants were interviewed individually using the Zoom videoconferencing platform. They were asked to discuss potential benefits and drawbacks surrounding the depiction of HCCAs as health professionals. Interviews were transcribed verbatim and uploaded to NVivo (version 12; QSR International, Inc) for thematic analysis.

**Results:**

Twenty-four health professionals participated in the study (19 women, five men; *M* age = 42.75 years, *SD* = 10.71). Three themes were developed from their interview data. Participants said that portraying HCCAs as health professionals is a form of misrepresentation and may mislead program users. Participants were also concerned that these depictions could draw from stereotypes regarding the appearance of health professionals, which might affect people's expectations surrounding these programs or their willingness to use them. Despite these concerns, some participants thought that there may be benefits to depicting HCCAs as health professionals, particularly in terms of providing a sense of reassurance to people seeking health support.

**Conclusions:**

The health professionals in this study expressed mixed views toward the depiction of HCCAs as health professionals. Their insights may prompt further discussion on the appropriate depiction of HCCAs among developers and other stakeholders.

## 1 Introduction

Health professionals play an important role in the promotion and management of people's health and wellbeing. They support people through a variety of activities, such as diagnosing and treating health conditions, delivering long-term chronic care, and encouraging healthy behaviors and lifestyle habits (1, 2). Moreover, their compassion and understanding can provide individuals with reassurance at a time when they are feeling particularly vulnerable and uncertain about their health. Generally speaking, the public recognizes the knowledge and expertise of health professionals and seems to value their guidance and support. For instance, research shows that people have high levels of trust in health professionals (3, 4) and view them as a reliable source of health information (5, 6). Studies also show that people have considerable admiration and respect for those working in the health professions (7, 8).

Public sentiment toward health professionals might be leveraged to improve the appeal of certain health technologies. Consider health care conversational agents (HCCAs), automated software programs that provide health-related support through a conversational interface (e.g., health chatbots) (9, 10). In recent years, researchers and companies have developed HCCAs to address a wide range of health issues and concerns (10–13). Some of these programs are designed to simulate health professionals in terms of their presentation or appearance (14). For instance, an HCCA might be given a health professional title in its name (15, 16), and an HCCA with a virtual or graphical body may be dressed in a health professional uniform (17, 18). Previous research has shown that the public prefers HCCAs that are depicted as health professionals over those that appear in nonprofessional roles (19). This finding can likely be attributed to the fact that these depictions promote feelings of confidence and trust in users (20, 21).

Although the public seems to have favorable views toward the depiction of HCCAs as health professionals, the views of health professionals are less clear. Health professionals have considerable experience with the delivery of health services and may be able to draw on their expertise to provide novel insight into the use of these depictions. Their insight into potential ethical or legal issues would be particularly valuable. The purpose of the current study was to learn more about this topic. Health professionals with a variety of professional backgrounds were recruited and asked to share their views on the depiction of HCCAs as health professionals. Their comments were expected to offer guidance on whether and how these depictions should be used in the provision of health care.

## 2 Methods

This study is part of a larger project exploring health professionals' views toward HCCAs. The methods are described in greater detail in a previous article (22). The results reported in the current article are distinct from those reported in the previous article.

### 2.1 Study design

This study used a qualitative descriptive design. The aim of qualitative description is to provide a rich and detailed description of a phenomenon, emphasizing surface readings of the available data to preserve participants' voices and perspectives (23, 24). Study data were collected using cross-sectional semistructured interviews.

### 2.2 Participants and recruitment

This study included Canadian health professionals, specifically physicians, nurses, and regulated mental health professionals. Participants were recruited through social media posts, classified websites, e-newsletters, and emails to relevant organizations. Our initial recruitment goal was 24 participants, eight in each of the three health professional groups. After these participants were recruited and interviewed, the interview data were assessed to determine the need for further participants. We felt that the dataset offered sufficient insight to address the research objective and anticipated that additional data would not alter the study's conclusions. Therefore, no additional participants were sought for the study. Participants received an entry into a draw for a $100 (Canadian) Amazon gift card for their participation.

### 2.3 Procedure

Participants were interviewed individually over the Zoom videoconferencing platform. Interviews were conducted between March and September 2021. Each participant was emailed a PDF document that provided basic information on HCCAs (delivery methods, input and output modalities, etc.) before their interview. This document included screenshots of several HCCAs (25–31), including HCCAs that simulate health professionals in terms of their presentation or appearance. The purpose of this document was simply to ensure that participants had a sufficient understanding of this technology prior to being interviewed. The interviews themselves began with personal introductions, a review of the study information, and the acquisition of verbal consent. Next, questions were administered using a semistructured interview format. In addition to broader questions on the use of HCCAs, participants were asked questions related to the depiction of HCCAs as health professionals. More specifically, they were asked to discuss potential benefits and drawbacks of depicting HCCAs as health professionals, as well as why these depictions should or should not be used. See Supplementary Material for the full list of interview questions. A debriefing was provided at the end of each interview.

### 2.4 Data analysis

A research assistant transcribed the interview recordings, removing any identifying information. The first author reviewed the transcripts for accuracy and uploaded the transcript files to NVivo (version 12; QSR International, Inc) for thematic analysis. The thematic analysis used a codebook approach and broadly followed the six phases described by Braun and Clarke (32, 33). To start, the first and second authors familiarized themselves with the dataset by reading and rereading the transcripts and making preliminary notes (phase one). Next, the same authors coded the dataset through an iterative process (phase two). They reviewed the transcripts of the first three interviews to generate preliminary codes and working definitions. The first author used this information to code the full dataset, creating additional codes when necessary. The second author independently coded 25% of the transcripts and found a high level of agreement with the first author (Cohen's *κ* = .81). The codes were refined to address the few disagreements, and the refined codes were applied to the full dataset. Next, the first and second authors generated initial themes (phase three) by clustering codes with similar patterns of meaning; further developed and reviewed the themes (phase four) by checking initial themes against both the coded data and the full dataset to ensure fit; and refined, defined, and named the themes (phase five) through further review and discussion. The first author wrote the original draft of the manuscript, and all authors contributed to subsequent revisions (phase six).

### 2.5 Trustworthiness

Several strategies were used to enhance the trustworthiness of the study, including investigator triangulation, the creation of an audit trail, purposive sampling of three distinct participant groups, the use of verbatim quotes to support the themes, and peer debriefing with digital health researchers in academia and the commercial sector. The data analysis was performed by two researchers with diverse professional backgrounds: one was primarily a quantitative researcher with a focus on digital health, whereas the other was primarily a qualitative researcher with a broader focus on health service delivery. The use of two researchers with varying backgrounds was expected to promote both analytical rigor and interpretive depth.

### 2.6 Ethics approval

This study received ethics approval from the research ethics board at the University of New Brunswick (009-2021).

## 3 Results

### 3.1 Participant characteristics

Twenty-four health professionals participated in the study: eight physicians, eight nurses, and eight regulated mental health professionals (two clinical psychologists, two psychotherapists, two counselors, and two clinical social workers). The sample consisted of 19 women and five men with a mean age of 42.75 years (*SD* = 10.71). Participants were from the Canadian provinces of Alberta, British Columbia, New Brunswick, Nova Scotia, Ontario, Prince Edward Island, and Saskatchewan. All participants had used conversational agents in the past, although only two had used HCCAs specifically.

### 3.2 Themes

We developed three themes from participants' interview data, broadly relating to misrepresentation, stereotyped portrayals, and reassurance. These themes are described in greater detail in the following subsections. Note that each of the three main participant groups—physicians, nurses, and regulated mental health professionals—offered discussion related to misrepresentation, stereotyped portrayals, and reassurance in their interviews. However, discussion related to stereotyped portrayals was more common among physicians than it was among nurses and regulated mental health professionals, whereas discussion related to misrepresentation was more common among the latter two groups than it was among physicians. No group differences were evident in discussion of reassurance.

#### 3.2.1 A form of misrepresentation

Participants suggested that depicting HCCAs as health professionals is a form of misrepresentation. They said that HCCAs lack the expertise and qualifications of real health professionals, and so portraying them as health professionals is inappropriate and potentially even fraudulent. In the words of one participant,

I think that's completely unethical in a way and fraud. You know, to say “I am a clinical psychologist” or “I'm a cardiologist” or “[I'm] a hematologist” when it's artificial. And yes, they can program lots of information and knowledge into it, but… the past experience, lived experience, experience that you have during your practicum and internships over the years of your schooling to enhance your practice, that's completely gone. (P4, psychotherapist)

People who engage with these depictions could mistakenly believe that they are receiving a similar standard of care as they would receive from a real health professional. This prospect concerned participants: “I wouldn't want to be giving the illusion that this is necessarily the same experience” (P14, psychotherapist). These depictions could also lead people to believe that they are interacting with a real health professional instead of an automated program, although this type of misunderstanding may be more likely to occur with some populations than others. For instance, one participant who works with vulnerable pregnant women said “I’ve worked with a lot of folks that are identified as special needs… They don’t even understand how they got pregnant, forget anything else. So they might think ‘That’s actually my nurse’” (P7, nurse). Such misunderstandings could have a negative impact on the credibility of health professionals and the health care system as a whole, particularly if a user misattributes the poor performance of an HCCA to a real care provider. One participant provided an example within a mental health context: “People will think they’re interacting with an actual human and then say ‘I tried counseling, and it didn’t work’” (P6, clinical social worker).

#### 3.2.2 Concern about stereotyped portrayals

Participants were concerned that the depiction of HCCAs as health professionals would draw from stereotypes regarding the appearance of health professionals. A stereotyped portrayal could trigger inappropriate or skewed expectations surrounding the conduct or behavior of the program, which might be counterproductive for care delivery. For instance, one participant discussed the appearance of a specific HCCA (shared in the preinterview document) that was depicted as a physician: “It is stereotypical, in terms of the typical lab coat and whatnot. And it can lead to patients having… ideas in advance about what the physician should say or how they should guide you” (P12, physician). Some participants were also concerned about health professional depictions that display particular demographic characteristics, especially characteristics that reinforce stereotypes about the sex, gender, and race or ethnicity of health professionals. Such portrayals could elicit negative responses from certain populations and create barriers to use. For example, one participant was worried about HCCAs that are portrayed as the traditional “White male doctor,” saying “Is that representative of the demographic that's accessing those supports? If I'm an Indigenous woman, I might be less inclined to use that, you know?” (P7, nurse). In their interviews, participants emphasized the fact that stereotyped portrayals are not an accurate reflection of many health professionals and fail to capture the diversity of real providers. As one participant said, “It's not cookie-cutter… The reality is that physicians and nurses and health care team members all have a variety of different appearances” (P22, physician). Another participant reinforced this point with respect to uniforms specifically, saying “You can't just pick one generic doctor or nurse, right? Everyone dresses differently” (P19, nurse).

#### 3.2.3 A sense of reassurance

Despite their concerns over misrepresentation and stereotyped portrayals, participants said that depicting HCCAs as health professionals could be useful for providing a sense of reassurance to people who are seeking health support. For instance, participants said that these depictions might promote feelings of comfort in program users, providing them with the feeling that they are interacting with a provider who genuinely cares about their health instead of an automated technology. As one participant said, “I think it might be a truer human-to-human experience. I could certainly see how the patient might feel a bit more comfortable with that rather than a perceived computer or robot” (P22, physician). Another participant reiterated this point, noting that these depictions may be particularly beneficial for certain demographic groups, such as older adults: “It would give them [patients] someone that they're a little more comfortable talking to. Particularly older patients who would feel very awkward and uncomfortable talking to a screen” (P13, physician). Participants also said that depicting HCCAs as health professionals could promote feelings of trust in users. Greater trust could translate into better adherence to the content and recommendations supplied by the HCCA, which would be beneficial provided the program is offering sound, appropriate advice. As one participant commented, “I definitely think that people would sort of follow recommendations and be more apt to trust and believe in the messages being sent” (P12, physician). Another participant emphasized this same point by imagining the perspective of a patient when interacting with this type of program: “This is a fake nurse, but you know, I think I can trust it because it's got the appearance of a nurse” (P7, nurse).

## 4 Discussion

### 4.1 Principal findings

Participants in this study expressed concern about the depiction of HCCAs as health professionals. They said that portraying HCCAs as health professionals is a form of misrepresentation and may mislead program users. They were also concerned that these depictions could draw from stereotypes regarding the appearance of health professionals, which might affect people's expectations surrounding these programs or their willingness to use them. Some participants thought that there may be benefits to depicting HCCAs as health professionals, particularly in terms of providing a sense of reassurance to people who are seeking health support. However, most participants focused their discussion on the drawbacks of these depictions and suggested that they should be used cautiously, if at all.

### 4.2 Comparison with prior work

Previous findings on HCCAs support our participants' concerns surrounding misrepresentation. For instance, there is a large body of literature on the limited capabilities of HCCAs (34–36), which reinforces our participants' claims that these programs lack the expertise and qualifications of real health professionals. There is also support for participants' assertion that depicting HCCAs as health professionals could mislead users with respect to the capabilities or the artificial nature of these programs. More specifically, research has shown that HCCAs with a health professional appearance are seen as more credible than those with a casual or informal appearance (20), despite the fact that these two types of programs would have the same limitations and constraints. Moreover, there is evidence that people can experience confusion or misunderstanding over whether HCCAs are real care providers, even after they are explicitly told that these programs are not actual providers (21, 37). This phenomenon is not limited to HCCAs that simulate health professionals, but these types of portrayals would likely increase the potential for confusion or misunderstanding in users.

In addition to their concerns over misrepresentation, the participants in our study were worried that the depiction of HCCAs as health professionals would draw from stereotypes regarding the appearance of health professionals. These stereotyped portrayals could have drawbacks in terms of people's expectations surrounding these programs or their willingness to use them. To our knowledge, there have been no studies with a specific or dedicated focus on the drawbacks of these portrayals. However, some researchers have employed stereotyped portrayals when investigating other outcomes, and this research may provide insight into potential issues surrounding their use. For instance, participants in one study reported that a chatbot conforming to the White male doctor stereotype was less warm and a less satisfying communication partner than a chatbot depicted as a White female doctor (38). Notably, this difference emerged even though the dialogue in the two chatbots was essentially identical. The study in question was examining gender bias rather than stereotyped portrayals specifically, and so it is not possible to make any definitive claims about the impact of these portrayals based on the results. Regardless, such findings are consistent with the idea that stereotyped portrayals might affect user engagement and, in some cases, deter program use.

In terms of benefits, the participants in our study said that depicting HCCAs as health professionals might provide users with a sense of reassurance, particularly feelings of comfort and trust. This suggestion is consistent with previous research conducted with the general public, which has also indicated that these depictions can increase comfort and trust among users (20, 21). Although applying health professional titles or uniforms to HCCAs is a somewhat trivial design choice from a technical standpoint, it seems to be sufficient to improve people's perceptions of this technology. Increased reassurance in HCCAs could facilitate greater use of and adherence to these programs, which might benefit program users and the health care system more broadly. However, any benefits would be contingent on this technology offering appropriate guidance and advice. Given the limitations and constraints of HCCAs, there could be situations in which greater reassurance in these programs is not warranted.

### 4.3 Differences between health professionals

Some of the results varied between the different types of health professionals who participated in the study. Although the reasons for this variation are not immediately clear, it is possible to make some informed speculations based on past research. To start, physicians were more likely than nurses and regulated mental health professionals to express concern over stereotyped portrayals. The use of stereotypical physician attire (e.g., white lab coats) in real health care settings has received a great deal of attention in recent years (39, 40), and there has been some discussion on the extent to which this attire should be used (41–43). There has also been much discussion on diversity among physicians and challenges faced by those who do not fit the “White male doctor” stereotype (44–47). The increased focus on these and similar issues in real health care settings may have made the physicians in our study sensitive to the use of stereotyped portrayals with HCCAs.

Meanwhile, physicians were less likely than nurses and regulated mental health professionals to discuss topics related to misrepresentation. Nurses and regulated mental health professionals tend to have less occupational power and prestige than physicians (48–52), such that they may rely more on titles, uniforms, and similar identifiers to assert their professional authority. The appropriation of these identifiers by technologies such as HCCAs could be seen as an encroachment on their professions that conflicts with their ongoing efforts to obtain greater recognition and perceived legitimacy in health settings. Nurses and regulated mental health professionals also tend to place a greater emphasis on the mental and emotional aspects of care than physicians, who are typically more focused on evaluation, diagnosis, and treatment (53, 54). This difference in focus may explain why the nurses and mental health professionals in our study were particularly concerned about confusion or misunderstanding in individuals who use HCCAs that simulate health professionals. In the future, researchers should explore our findings in greater detail to gain a better understanding of health professional differences in this area, with a particular focus on learning more about the reasons for these differences.

### 4.4 Practical implications

The results of this study may be informative for researchers and companies that are considering a health professional presentation or appearance for their HCCAs. On one hand, depicting HCCAs as health professionals might encourage a sense of reassurance in individuals. Greater reassurance could increase use of and adherence to HCCAs, which has the potential to benefit program users and the health care system more broadly. On the other hand, there are certain ethical and legal issues concerning misrepresentation that require serious consideration. For instance, there could be legal consequences for developers if the appearance or presentation of an HCCA misleads users with respect to the capabilities or the artificial nature of the program. It is also worth noting that the use of health professional titles is often legally restricted to fully trained and qualified care providers (55–57), and attaching these titles to technologies that provide health-related support to the public might be seen as a form of misuse. In addition, developers should be aware of potential issues surrounding stereotyped portrayals and the impact that these portrayals could have on individuals. For instance, portraying an HCCA as a traditional White male doctor could be a barrier to use for some populations and may limit adoption of this technology. Given the increased availability and use of HCCAs in recent years, more discussion is needed among developers and other stakeholders (policymakers, practitioners, etc.) to produce clearer and more concrete guidelines regarding the appropriate depiction of these programs.

### 4.5 Limitations

This study had several limitations. First, many of the health professionals who participated had never used an HCCA, and by implication had no hands-on experience with HCCAs that simulate health professionals. Although they were shown several examples of these types of programs prior to being interviewed, their views might have differed had they greater experience with these depictions. Second, participation was restricted to health professionals in Canada, whose views were likely shaped by their experiences within Canadian health care settings. The findings may not be transferable to health professionals in other countries, where approaches to professional regulation and health care delivery may differ. Third, most of the study participants (79%) identified as women, which likely reflects the fact that women occupy a majority of the health care positions in Canada (58, 59). Having more men in the sample might have resulted in additional insights.

## 5 Conclusion

The health professionals in this study had mixed views toward the depiction of HCCAs as health professionals. Although they thought that depicting HCCAs as health professionals might provide people with a sense of reassurance, they were concerned about potential issues surrounding misrepresentation and stereotyped portrayals. More discussion about these depictions among stakeholders could help clarify their acceptability and provide further guidance for their use in health settings.

## Data Availability

The dataset discussed in this article is not publicly available because it consists of interview transcripts. Data access is restricted to safeguard the privacy and confidentiality of the participants. Requests to access the dataset should be directed to luke.macneill@unb.ca.
