# Correction: Exploring health professionals' views on the depiction of conversational agents as health professionals: a qualitative descriptive study

**DOI:** 10.3389/fdgth.2025.1718775

**Published:** 2025-11-03

**Authors:** A. Luke MacNeill, Lillian MacNeill, Alison Luke, Shelley Doucet

**Affiliations:** ^1^ Centre for Research in Integrated Care, University of New Brunswick, Saint John, NB, Canada; ^2^ Department of Nursing and Health Sciences, University of New Brunswick, Saint John, NB, Canada

**Keywords:** conversational agents, chatbots, health care, health professionals, qualitative, interview, views

**A Correction on**
Exploring health professionals' views on the depiction of conversational agents as health professionals: a qualitative descriptive study By MacNeill AL, MacNeill L, Luke A and Doucet S (2025). Front. Digit. Health 7:1590514. doi: 10.3389/fdgth.2025.1590514

In the published article, some research findings were incorrectly identified as anecdotal evidence. In the same sentence, the discussed research findings aligned with one citation but not the other.

A correction has been made to Discussion, Comparison with Prior Work, paragraph 1, to address both points. The sentence in question previously stated:

“Moreover, there is some anecdotal evidence that people interacting with HCCAs can mistake them for real health professionals, even after they are explicitly told that these programs are not actual providers (21, 37).”

The corrected sentence appears below:

“Moreover, there is evidence that people can experience confusion or misunderstanding over whether HCCAs are real care providers, even after they are explicitly told that these programs are not actual providers (21, 37).”

The change does not affect the scientific conclusions of the article.

The original article has been updated.

